# Effect of Mobile Phone Text Message Reminders on the Completion and Timely Receipt of Routine Childhood Vaccinations: Superiority Randomized Controlled Trial in Northwest Ethiopia

**DOI:** 10.2196/27603

**Published:** 2021-06-15

**Authors:** Zeleke Abebaw Mekonnen, Kassahun Alemu Gelaye, Martin Were, Binyam Tilahun

**Affiliations:** 1 Department of Health Informatics, Institute of Public Health University of Gondar Gondar Ethiopia; 2 Department of Epidemiology and Biostatistics, Institute of Public Health University of Gondar Gondar Ethiopia; 3 Department of Biomedical Informatics Vanderbilt Medical Center Nashville, TN United States

**Keywords:** mHealth, eHealth, mobile phone, text message, short message service, reminder, immunization, vaccination, Ethiopia

## Abstract

**Background:**

Nonattendance at vaccination appointments is a big challenge for health workers as it is difficult to track routine vaccination schedules. In Ethiopia, 3 out of 10 children have incomplete vaccination and the timely receipt of the recommended vaccines is low. Thus, innovative strategies are required to reach the last mile where mobile technology can be effectively utilized to achieve better compliance. Despite this promising technology, little is known about the role of text message–based mobile health interventions in improving the complete and timely receipt of routine childhood vaccinations in Ethiopia.

**Objective:**

This trial aimed to determine the effect of mobile phone text message reminders on the completion and timely receipt of routine childhood vaccinations in northwest Ethiopia.

**Methods:**

A two-arm, parallel, superiority randomized controlled trial was conducted in 9 health facilities in northwest Ethiopia. A sample size of 434 mother-infant pairs was considered in this trial. Randomization was applied in selected health facilities during enrollment with a 1:1 allocation ratio by using sealed and opaque envelopes. Participants assigned to the intervention group received mobile phone text message reminders one day before the scheduled vaccination visits. Owing to the nature of the intervention, blinding of participants was not possible. Primary outcomes of full and timely completion of vaccinations were measured objectively at 12 months. A two-sample test of proportion and log-binomial regression analyses were used to compare the outcomes between the study groups. A modified intention-to-treat analysis approach was applied and a one-tailed test was reported, considering the superiority design of the trial.

**Results:**

A total of 426 participants were included for the analysis. We found that a higher proportion of infants in the intervention group received Penta-3 (204/213, 95.8% vs 185/213, 86.9%, respectively; *P*<.001), measles (195/213, 91.5% vs 169/213, 79.3%, respectively; *P*<.001), and full vaccination (176/213, 82.6% vs 151/213, 70.9%, respectively; *P*=.002; risk ratio 1.17, 95% lower CI 1.07) compared to infants in the usual care group. Similarly, a higher proportion of infants in the intervention group received Penta-3 (181/204, 88.7% vs 128/185, 69.2%, respectively; *P*<.001), measles (170/195, 87.1% vs 116/169, 68.6%, respectively; *P*<.001), and all scheduled vaccinations (135/213, 63.3% vs 85/213, 39.9%, respectively; *P*<.001; risk ratio 1.59, 95% lower CI 1.35) on time compared to infants in the usual care group. Of the automatically sent 852 mobile phone text messages, 764 (89.7%) were delivered successfully to the participants.

**Conclusions:**

Mobile phone text message reminders significantly improved complete and timely receipt of all recommended vaccines. Besides, they had a significant effect in improving the timely receipt of specific vaccines. Thus, text message reminders can be used to supplement the routine immunization program in resource-limited settings. Considering different contexts, studies on the implementation challenges of mobile health interventions are recommended.

**Trial Registration:**

Pan African Clinical Trial Registry PACTR201901533237287; https://pactr.samrc.ac.za/TrialDisplay.aspx?TrialID=5839

## Introduction

### Background

Vaccine-preventable diseases (VPDs) remain a common cause of childhood mortality with an estimated 3 million deaths globally each year. Vaccines have proven to be one of the most effective preventive interventions and they provide successful means for controlling VPDs [1-4]. The World Health Organization estimates that 29% of deaths in children younger than 5 years can be averted with existing vaccines [5]. Evidence also shows that being fully vaccinated is associated with 22% lower mortality in children [6]. Improving vaccination coverage is a priority for global health [7,8], and timeliness is an essential component of child vaccination that reduces susceptibility to VPDs [9]. The World Health Organization recommends that vaccines must be given within specified vaccination schedules and intervals [10] although there is no agreed upon definition of timeliness of vaccination across different countries [11]. Vaccine effectiveness also depends on the timing of its administration [12,13].

Despite the tremendous efforts to improve immunization programs, vaccination coverage and timeliness have remained suboptimal [14-16]. Globally, in 2018, coverage of the third dose of a vaccine protecting against diphtheria, tetanus, and pertussis remained at 86% [17]. Improving child vaccination coverage in low-income countries is challenging, and the vaccination coverage in Africa has been reported to be stalled at 76% [17]. Reports also show that globally, 5.9 million children were partially vaccinated in 2018 leaving many children susceptible to VPDs [18]. In low-income countries, failure to attend vaccination appointments is still a significant problem to health care providers, thereby contributing to delayed and missed vaccinations [12,19,20]. Studies in low-and-middle-income countries have also shown that despite relatively high vaccination coverage, there is a gap in the timeliness of childhood immunization [21-26].

In Ethiopia, routine childhood vaccines are administered at birth, fourth week, sixth week, tenth week, and ninth month before 1 year of age [2]. However, the completeness and timeliness of vaccination are not optimal at the national level [27,28]. The Ethiopian Demographic and Health Survey 2016 report indicates that only 39% of children received all the basic vaccinations [29]. Besides, the 2019 mini Ethiopian Demographic and Health Survey reported that the full vaccination coverage has reached 43%, with a steady rise in vaccination coverage over time [30]. In terms of vaccination completion, Ethiopia has the second largest number of incompletely vaccinated children in Africa, after Nigeria [31]. A systematic review and meta-analysis on incomplete vaccination in Ethiopia also reported that 3 out of 10 children have incomplete vaccination [32]. Similarly, studies in different parts of the country revealed that the partial vaccination coverage ranged from 17% to 45.5% [33-37]. Moreover, only a small proportion of children are vaccinated on time [28,38]. According to the national immunization coverage survey report, the prevalence of valid vaccination doses for all vaccines was only 18.6% [39]. In this study setting, the baseline assessment also indicated that full vaccination coverage was 64.3% and the timely completion for all vaccines was only 31.9% [40]. Consequently, the targets set for the different vaccines are not met [28,30].

Maintaining reductions in mortality due to VPDs relies upon continued vaccination uptake that is reliant on subsequent attendance for each vaccination appointment. However, the frequently mentioned reasons for missed and delayed vaccination in children are related to the difficulty in tracking the vaccination appointments as scheduled [13,19,28,38,41]. Forgetfulness and being unaware of the need to return for subsequent doses were among the common reasons for nonattendance to vaccination schedules, which are remediable through appropriate reminder services [28,37,38,40,42-47]. Studies have shown that achieving universal coverage with all recommended vaccines requires tailored strategies that address barriers to vaccination [15,16,28,48-50]. Thus, innovative strategies are required to reach the last mile where mobile technology can be effectively utilized to augment the immunization program. Globally, the rapid development of mobile technology has created new ways for addressing public health challenges where mobile phones are gradually becoming an integral part of health care services worldwide [51]. Mobile health (mHealth) is the use of mobile phone technology to deliver health care [52]. Among the mobile phone features, SMS remains one of the most popular forms of mobile communication with the potential to reach a large number of individuals at relatively low cost [53-55]. The World Health Organization also reported that SMS is the most common mobile phone feature used for appointment reminders to strengthen the health care system [52].

Different studies have shown that text message reminders are promising in improving attendance at health facilities and health outcomes [47,56-61]. Several studies have suggested that parental reminders for vaccination should include modern technologies such as mobile phone text message reminders [62-65]. However, the effectiveness of mobile phone–based text message reminders in improving the immunization program varies between settings. We identified studies that examined mobile phone text message reminders for vaccination, some of which found it effective in increasing vaccination uptake [44,62,66-72] while the other studies found no statistically significant effect on child vaccination [73-77]. Thus, the scientific body of evidence on the current practice in low-income countries is still limited [14,63,72,77-79].

### Objectives

In Ethiopia, mobile phone technology access is expanding rapidly [80,81]. Despite this promising and fast-growing technology, little is known about the possible role of mHealth interventions in improving the immunization program in the Ethiopian local context. Hence, this trial was conducted to assess the effect of mobile phone text message reminders in improving child vaccination service uptake in Gondar city, northwest Ethiopia.

## Methods

### Trial Design

This study applied a two-arm, parallel, superiority, individually randomized controlled trial design with a 1:1 allocation ratio. This trial followed a published study protocol that details the methods and approaches implemented in the trial [82]. This randomized controlled trial was conducted in the public health facilities (8 health centers and 1 comprehensive specialized hospital) of Gondar city, northwest Ethiopia between May 2019 and June 2020. This city has an estimated total population of 390,644 of which 12,149 are younger than 1 year [83]. The baseline assessment in the study area indicated that 78.9% (360/456) of the mothers had the intention to use mobile phone text message reminders for child vaccination. The assessment also pointed out that among mothers who owned a mobile phone, 91.0% (415/456) can read text messages, which makes the trial feasible in the study setting [84].

### Participants

For this particular study, eligible mother-infant pairs from the University of Gondar Comprehensive Specialized Hospital and all the 8 health centers were included. The eligibility criteria for the study participant selection are shown in Textbox 1.

**Inclusion and exclusion criteria for the participants in this study.**

**Inclusion Criteria**

- Mothers who have an infant who took the Bacillus Calmette-Guérin vaccination up to 4 weeks of age
- For twin infants presented for vaccination, the younger infant was included
- Mothers aged 18 years and older
- Mothers who had a working mobile phone
- Resided in the study area at least for 6 months (permanent residents)
- Mothers who were willing to provide consent for participation in the study

**Exclusion Criteria**

- Infants who already received vaccinations other than Bacillus Calmette-Guérin or polio zero vaccines
- Mothers who could not read mobile phone text messages in Amharic or English languages
- Mothers who had no mobile network access in their house/compound
- Mothers who planned to relocate out of the study area during the study follow-up period

During enrollment, when more than one eligible mother-infant pair from the same house/compound presented to the health facility for vaccination, only one infant (younger) was included to reduce the risk of information pollution among study participants. The recruitment of the study participants started on May 1, 2019 and ended on June 26, 2019. Study participants were followed up for 12 months.

### Intervention

Participants assigned to the intervention group received the routine vaccination appointment reminder and additional mobile phone text message reminders one day before the scheduled vaccination visits on the sixth week, tenth week, fourteenth week, and ninth month after childbirth. Participants in the usual care group used vaccination cards and were informed of the due date of the next vaccination schedule verbally by health care providers working in health facilities during the facility visit. For the intervention group, a computerized text message reminder system was designed and developed for this particular study considering the local context by the eHealthLab Ethiopia research team [85]. The computerized text message reminder system has 2 components: a web-based application for client registration and automatic reminder scheduling (Multimedia Appendix 1) and a mobile SMS app for sending appointment reminders (Multimedia Appendix 2). The mobile phone text message reminders were designed and developed based on literature search, consultation with experts, and considering users’ preferences. Based on the findings from baseline assessments in the study area on mothers’ intentions and preferences to use text message reminders [84], mobile phone text messages were developed and delivered to users both in Amharic and English languages (Multimedia Appendix 2). The detailed development process of the automated reminder system and its function is described in a separate publication [85].

### Outcomes

Full vaccination coverage and on-time full vaccination coverage were the primary outcomes for this trial. The secondary outcomes included coverage and timeliness for specific vaccine doses. In this trial, an infant was defined as having achieved full (complete) vaccination once he/she had received all the recommended vaccines included in the national immunization schedule, namely, a dose of Bacillus Calmette-Guérin, 3 doses of oral polio vaccine, 3 doses each of pentavalent and pneumococcal conjugate vaccine, 1 dose of inactivated polio vaccine, 2 doses of rotavirus vaccine, and 1 dose of measles vaccine by the age of 12 months [2,3,29,36]. Further, the timeliness of vaccine administration (age-appropriate vaccination) was determined based on the routine schedule of Ethiopia’s national immunization program–recommended age of vaccination. Accordingly, on-time vaccination for specific vaccines was defined as a specific vaccine dose administered within 4 days before the due date [86-89] and within 4 weeks (inclusive of the due date) after the recommended age specified in the national immunization schedule [9,10,41,87-91]. The denominator for the timeliness of specific vaccines was considered from those infants vaccinated for that specific vaccine [9,10,41,87-91]. In addition, on-time full vaccination was defined as all vaccine doses administered within 4 days before the due date [86-89] and within 4 weeks (inclusive of the due date) after the recommended age specified in the national immunization schedule [19,41,87,90-93]. Otherwise, it was considered as not fully vaccinated on time if at least one vaccine dose was given early, late, or missed at all at the end of the follow-up. The denominator for on-time full vaccination considered all infants who were enrolled for this trial [19,41,87,90-93].

### Study End Points

The effect of mobile phone text message reminders was measured by comparing the difference in the vaccination coverage and timely receipt of vaccination between the intervention and usual care groups at 6 weeks, 10 weeks, 14 weeks, 9 months, and 12 months of age.

### Sample Size Determination

Before the commencement of the randomized controlled trial, a baseline assessment was conducted in the study area to obtain recent estimates on the complete and timely vaccination status of children [40]. The sample size required for this trial was determined using the STATA statistical software (Release 14. College Station, TX: StataCorp LP). The sample size was calculated for both vaccination completeness and timeliness independently. Finally, the larger sample size was considered for this trial.

#### Sample Size Determination Using Full Vaccination Coverage

The sample size calculation for full vaccination assumed a power of 90%, a significance level (α) of 5% for superiority design, a difference of 15% between the intervention and usual care groups from the baseline full vaccination coverage of 64.3% [40] to 79.3%, and 20% for lost to follow-up with intervention:control ratio of 1:1. With these assumptions, the sample size required for both study groups was 366 (183 in each group).

#### Sample Size Determination Using On-Time Full Vaccination Coverage

A sample size of 434 mother-infant pairs (217 in each of the intervention and usual care groups) was required to detect a difference of 15% between the intervention and usual care groups from the baseline on-time full vaccination coverage of 31.9% [40] to 46.9%, 90% power, significance level (α) of 5% for superiority design, and accounting 20% for lost to follow-up. Therefore, for the 2 arms, the final sample size of the trial was the larger sample size of 434 (217 in the intervention group and 217 in the usual care group).

### Randomization

The units of randomization were mother-infant pairs randomized in one of the two study arms. All mother-infant pairs who were eligible and gave informed written consent for participation were randomly assigned to either of the study groups by using a simple randomization technique. Randomization was applied in the selected health facilities during enrollment with a 1:1 allocation ratio by using sealed and opaque envelopes within each health facility separately.

### Allocation Concealment

To prevent foreknowledge of intervention assignment, we used identical and small-sized sealed opaque envelopes for the random sequence generation. Random sequence generation was ensured by a research assistant ahead of the study subject’s enrollment with which study arms were marked on paper and folded to fit the envelope. Finally, the sealed envelopes prepared for both the intervention and usual care groups were combined for each health facility and shuffled so that the allocation sequence remained concealed. When the mother-infant pairs were found to be eligible for the trial, they were assigned to the intervention or usual care group by randomly picking up a closed envelope.

### Blinding

Owing to the nature of the intervention, blinding of the study participants was not possible. Health workers who administered the vaccines and recorded the vaccination status of the infants were blinded to study group allocations. Outcome assessors were also blinded to the intervention allocation.

### Implementation

This trial was implemented as per the a priori published protocol [82]. Initially, the eligibility of the mother-infant pairs presenting for Bacillus Calmette-Guérin vaccination of an infant at the vaccination units of the included health facilities was assessed. During enrollment, informed written consent was obtained from each eligible study participant. Enrollment and assignment of the intervention to the study participants were made by trained data collectors. In addition, baseline information was collected from both intervention and usual care groups on sociodemographic characteristics, health care service use, and mobile phone use characteristics by using validated data collection tools. All mothers assigned to the intervention group were assigned a unique code and provided orientation on how to read mobile phone text messages received from the automated reminder system. Thereafter, participants assigned to the intervention group received mobile phone text message reminders sent from the automated text message reminder system one day before the due date of the scheduled vaccinations. A research assistant was assigned to manage the automated reminder system throughout the follow-up period. Data for primary and secondary outcomes were collected from written vaccination records found in the health facility’s expanded program on immunization (EPI) registers by trained outcome assessors. During the follow-up, data on the vaccination status of infants were collected regularly. Validated data collection tools were used to collect outcome data [40].

### Statistical Analysis

The baseline data collected from the study participants and the outcome data collected from EPI registers were entered into the EpiData version 3.1 software (EpiData Association). Finally, the data were exported to the STATA statistical software for analysis. Analyses were done with the modified intention-to-treat analysis principle at the participant level so that all randomized participants with available outcome data were included for the analysis, regardless of the degree of exposure to study intervention [94]. Initially, descriptive statistics were computed. The household wealth index was created by principal component analysis, including variables on asset ownership, housing characteristics, ownership of animals, and farming [95]. For assessing the comparability of the data on the baseline characteristics of the study participants randomized to the intervention and usual care groups, chi-square tests were performed. Proportions were used to present the vaccination coverage and timeliness for each vaccine. To assess the changes in the vaccination status between the 2 study groups, absolute differences in the proportions with corresponding 95% CIs were computed. A two-sample test of proportion was applied to assess the statistical significance of the absolute differences. Further, risk ratios (RRs) for primary outcomes were assessed and compared between the intervention and the usual care groups by using log-binomial regression analysis [96]. As an additional analysis, post hoc subgroup analysis was also considered to assess the heterogeneity of the treatment effects by subgroups of sociodemographic characteristics. For the subgroup analysis, the log-binomial model was applied and interaction terms were tested accordingly to explore potential effect modification. Taking into account the superiority design of the trial, one-tailed test was performed and reported [97]. The effect was expressed as RR with a corresponding 95% lower confidence interval. A significance level of .05 was considered for this study. Finally, a post hoc study power analysis was conducted. The conduct, analysis, and reporting of results were done in accordance with the CONSORT updated guidelines for reporting parallel group randomized trials. In addition, the trial was reported in accordance with the CONSORT-EHEALTH checklist (Multimedia Appendix 3) [98].

### Ethics and Confidentiality

This study obtained ethical approval from the University of Gondar Institutional Ethical Review Board Reference O/V/P/RCS/05/060/2018. The purpose and procedures of the trial were explained to all the study participants. Accordingly, informed written consent was sought from all the study participants during enrollment. Confidentiality and anonymity of the information obtained were maintained at all levels of data handling. Before the commencement of the study, permission was obtained from all the required administrative levels, including the Amhara Public Health Institute and Ethio telecom.

## Results

### Enrollment of the Study Participants

**Figure 1:**
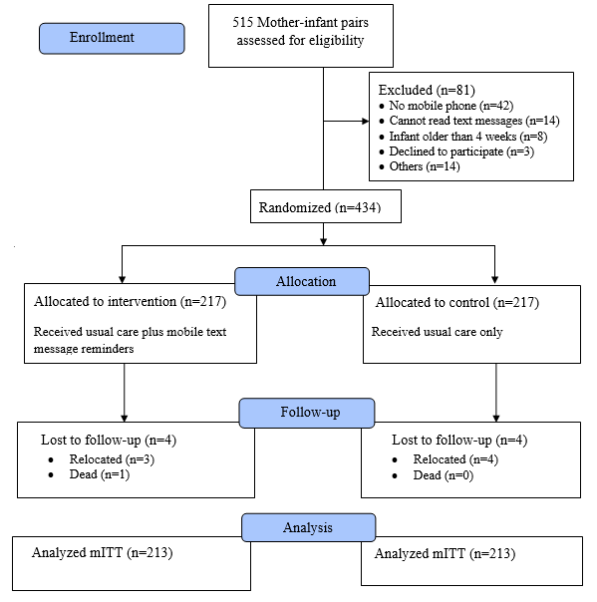
The CONSORT flow diagram of study participant enrollment, randomization, allocation, and analysis for the trial in Gondar city, northwest Ethiopia in 2020 (N=426). mITT: modified intention-to-treat.

Between May 1, 2019 and June 26, 2019, 515 mother-infant pairs were assessed for eligibility. Accordingly, 434 eligible mother-infant pairs were enrolled, with 81 excluded for not meeting the eligibility criteria (Figure 1). Of the 434 mothers enrolled, 217 were assigned to the intervention group and 217 to the usual care group. Seven participants relocated from the study area and there was 1 natural death of an infant during the study period, which actually had no relation with the outcome of interest. Thus, the analytic sample consisted of 426 mother-infant pairs who completed a 12-month follow-up in both groups with the modified intention-to-treat analysis principle (Figure 1).

### Characteristics of the Study Participants

The baseline characteristics of both the study groups are shown in Table 1. To validate the randomization, the characteristics of the mothers and infants of the usual care and intervention groups were examined. At baseline, the characteristics of the study participants were similar between usual care and intervention groups. The chi-square test for the baseline characteristics indicated that there was no statistically significant difference between the usual care and intervention groups at the start of the trial (Table 1).

**Table 1:** Baseline characteristics of the study participants enrolled in the trial in Gondar city, northwest Ethiopia in 2020 (N=426).

Characteristics		Intervention group (n=213), n (%)	Usual care group (n=213), n (%)	*P* value
**Mother’s age (years)**				.42
	≤24	64 (30.1)	71 (33.3)	
	25-34	123 (57.7)	110 (51.7)	
	≥35	26 (12.2)	32 (15.1)	
**Marital status**				.86
	Currently married	195 (91.5)	194 (91.1)	
	Currently not married	18 (8.5)	19 (8.9)	
**Religion**				.44
	Orthodox	180 (84.5)	181 (84.9)	
	Muslim	28 (13.1)	23 (10.8)	
	Others	5 (2.4)	9 (4.3)	
**Mother’s education**				.55
	No formal education	18 (8.4)	23 (10.8)	
	Primary	65 (30.5)	70 (32.9)	
	Secondary and above	130 (61.1)	120 (56.3)	
**Mother’s occupation**				.12
	Housewife	105 (49.3)	100 (46.9)	
	Employed	48 (22.5)	33 (15.5)	
	Merchant	38 (17.9)	49 (23.0)	
	Others	22 (10.3)	31 (14.6)	
**Sex of infant**				.77
	Female	107 (50.2)	110 (51.6)	
	Male	106 (49.8)	103 (48.4)	
**Birth order**				.16
	First	81 (38.1)	95 (44.6)	
	Second or later	132 (61.9)	118 (55.4)	
**Residence**				.53
	Rural	14 (6.6)	11 (5.2)	
	Urban	199 (93.4)	202 (94.8)	
**Household wealth index**				.30
	Poor	67 (31.5)	75 (35.2)	
	Middle	79 (37.0)	64 (30.1)	
	Rich	67 (31.5)	74 (34.7)	
**Family size**				.31
	<5 members	143 (67.1)	133 (62.4)	
	≥5 members	70 (32.9)	80 (37.6)	
**Distance to health facility**				.40
	<15 minutes	96 (45.1)	109 (51.2)	
	15-30 minutes	82 (38.5)	70 (32.9)	
	>30 minutes	35 (16.4)	34 (15.9)	
**Duration of mobile phone use**				.22
	≤2 years	37 (17.4)	47 (22.1)	
	>2 years	176 (82.6)	166 (77.9)	
**Mobile phone type**				.24
	Regular/standard	97 (45.5)	109 (51.2)	
	Smart	116 (54.5)	104 (48.8)	

### Effect of Mobile Phone Text Message Reminders on Full and Timely Completion of Vaccination

In this trial, the proportion of infants who completed the 12-month vaccination series in the intervention group was significantly higher than that of infants who completed the 12-month vaccination series in the usual care group (176/213, 82.6% vs 151/213, 70.9%, respectively; one-tailed *P*=.002). Similarly, comparisons in the timeliness of vaccination among the study groups revealed that timely completion of vaccination in the intervention group was significantly higher than that in the usual care group (135/213, 63.3% vs 85/213, 39.9%, respectively; one-tailed *P*<.001) (Table 2). Additionally, the timeliness of vaccination was compared between fully vaccinated infants in the intervention (n=176) and fully vaccinated infants in the usual care group (n=151). The findings showed that of those fully vaccinated infants in both groups, 135 (76.7%) in the intervention group and 85 (56.3%) in the usual care group were fully vaccinated on time.

**Table 2:** Effect of mobile phone text message reminders on full and timely completion of vaccination among infants in Gondar city, northwest Ethiopia in 2020 (N=426).

Vaccination status	Intervention group (n=213),vn (%)	Usual care group (n=213), n (%)	Absolute difference (%) (95% lower CI)^a^	*P* value^b^
Full vaccination	176 (82.6)	151 (70.9)	11.7 (5.1)	.002
On-time full vaccination	135 (63.3)	85 (39.9)	23.4 (15.7)	<.001

^a^One-tailed test reported for superiority design and there is no upper bound to the absolute difference.

^b^One-tailed test *P* value reported for the superiority design of the trial.

### Effect of Mobile Phone Text Message Reminders on Specific Vaccinations

The pentavalent and measles vaccination coverages were used to measure the specific vaccination coverage for the vaccine doses administered at the age of sixth week, tenth week, fourteenth week, and ninth month vaccination schedules. At the fourteenth week, Penta-3 coverage in the intervention group was significantly higher than that in the usual care group (204/213, 95.8% vs 185/213, 86.9%, respectively; one-tailed *P*<.001). At the ninth month, the proportion of infants who were vaccinated for measles in the intervention group was significantly higher than that of infants who were vaccinated for measles in the usual care group (195/213, 91.5% vs 169/213, 79.3%, respectively; one-tailed *P*<.001) (Table 3).

**Table 3:** Effect of mobile phone text message reminders on receipt of specific vaccines in Gondar city, northwest Ethiopia in 2020 (N=426).

Vaccination	Intervention group (n=213), n (%)	Usual care group (n=213), n (%)	Absolute difference (95% lower CI)^a^	*P* value^b^
Penta-1	210 (98.6)	203 (95.3)	3.3 (0.6)	.02
Penta-2	209 (98.1)	193 (90.6)	7.5 (3.9)	<.001
Penta-3	204 (95.8)	185 (86.9)	8.9 (4.5)	<.001
Measles	195 (91.5)	169 (79.3)	12.2 (6.7)	<.001

^a^One-tailed test reported for superiority design and there is no upper bound to the absolute difference.

^b^One-tailed test *P* value reported for the superiority design of the trial.

### Effect of Mobile Phone Text Message Reminders on Timely Receipt of Specific Vaccines

The trial showed that a significantly higher proportion of infants in the intervention group received Penta-3 vaccination on time as compared to infants in the usual care group (181/204, 88.7% vs 128/185, 69.2%, respectively; one-tailed *P*<.001). Similarly, a significantly higher proportion of infants in the intervention group also received measles vaccination on time as compared to infants in the usual care group (170/195, 87.1% vs 116/169, 68.6%, respectively; one-tailed *P*<.001) (Table 4).

**Table 4:** Effect of mobile phone text message reminders on timely receipt of specific vaccines in Gondar city, northwest Ethiopia in 2020.

Vaccination^a^	Intervention group, n (%)	Usual care group, n (%)	Absolute difference (95% lower CI)^b^	*P* value^c^
Penta-1	193 (91.9)	164 (80.8)	11.1 (5.6)	<.001
Penta-2	189 (90.4)	149 (77.2)	13.2 (7.3)	<.001
Penta-3	181 (88.7)	128 (69.2)	19.5 (12.9)	<.001
Measles	170 (87.1)	116 (68.6)	18.5 (11.5)	<.001

^a^Timeliness for the specific vaccines was calculated from vaccinated infants in both study groups. The total number of vaccinated infants for each vaccine is shown in Table 3.

^b^One-tailed test reported for superiority design and there is no upper bound to the absolute difference.

^c^One-tailed test *P* value reported for the superiority design of the trial.

### Log-Binomial Regression Analysis of the Intervention Effect on Primary Outcomes

Log-binomial regression analysis was performed to determine the effect of mobile phone text message reminders on the primary outcomes of full and timely completion of vaccination. Mothers in the intervention group were 17% more likely to fully vaccinate their infants as compared to mothers in the usual care group (RR 1.17, 95% lower CI 1.07). In addition, mothers in the intervention group were 59% more likely to timely complete all doses of vaccines for their infants as compared to mothers in the usual care group (RR 1.59, 95% lower CI 1.35) (Table 5). The potential impact of the text message reminders was also determined using attributable risk percent. The attributable proportion among those mothers who received mobile phone text message reminders indicated that 37.1% of infants’ on-time full vaccination could be attributed to the text message reminders.

**Table 5:** Log-binomial regression analysis of the effect of mobile phone text message reminders on the primary outcomes in Gondar city, northwest Ethiopia in 2020 (N=426).

Vaccination status	Intervention group (n=213), n (%)	Usual care group (n=213), n (%)	Risk ratio (95% lower CI)^a^
Full vaccination	176 (82.6)	151 (70.9)	1.17 (1.07)
On-time full vaccination	135 (63.3)	85 (39.9)	1.59 (1.35)

^a^One-tailed test reported for superiority design and there is no upper bound to the risk ratio.

### Subgroup Analysis of the Effect of Mobile Phone Text Message Reminders on Timely Completion of Vaccination

In this trial, subgroup analysis was performed to assess the effect of the mobile phone text message reminders within categories of subgroups of the sociodemographic characteristics and to evaluate for statistically significant subgroup differences. The interaction tests showed that there were no significant effect differences across the subgroups of the included sociodemographic variables, except for the household wealth index. With the interaction test, the effect differences across the subgroups of household wealth index were statistically significant (*P*=.01). The stratum-specific findings in the subgroup analysis by household wealth index showed that the intervention effects were significantly higher for mothers who belong to the middle or rich household wealth index group (RR 1.87, 95% lower CI 1.53). However, no significant difference was found in the mothers who belonged to the poor household wealth index group (RR 1.09, 95% lower CI 0.81) (Table 6).

**Table 6:** Subgroup analysis of the effect of mobile phone text message reminders on timely completion of vaccination in Gondar city, northwest Ethiopia in 2020.

Characteristics		Intervention group (n=135), n (%)	Usual care group (n=85), n (%)	Stratum-specific risk ratio (95% lower CI)^a^	*P* value^b^
**Mother’s age**					.46
	≤24 years	46 (71.9)	29 (40.8)	1.76 (1.35)	
	>25 years	89 (59.7)	56 (39.4)	1.51 (1.24)	
**Mother’s education**					.13
	Below secondary	44 (53.1)	25 (26.9)	1.97 (1.42)	
	Secondary and above	91 (70.0)	60 (50.0)	1.40 (1.17)	
**Household wealth index**					.01
	Poor	32 (47.8)	33 (44)	1.09 (0.81)	
	Middle or rich	103 (70.6)	52 (37.7)	1.87 (1.53)	
**Family size**					.46
	<5 members	89 (62.2)	55 (41.4)	1.51 (1.23)	
	≥5 members	46 (65.7)	30 (37.5)	1.75 (1.33)	
**Distance to health facility**					.61
	<15 minutes	63 (65.6)	47 (43.1)	1.52 (1.22)	
	≥15 minutes	72 (61.5)	38 (36.5)	1.68 (1.32)	
**Mobile phone type**					.39
	Regular/standard	55 (56.7)	43 (39.4)	1.44 (1.13)	
	Smart	80 (68.9)	42 (40.4)	1.71 (1.37)	

^a^One-tailed test reported for superiority design and there is no upper bound to the risk ratio.

^b^*P* value for the interaction term between intervention and baseline sociodemographic characteristics.

### Mobile Phone Text Message Delivery and Reading Status

**Figure 2:**
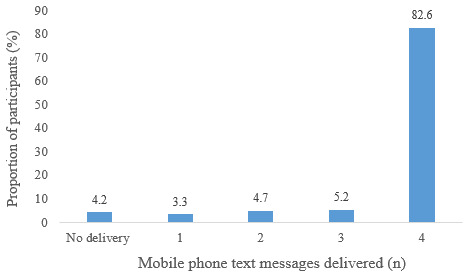
Mobile phone text message delivery status in the intervention group (n=213).

For the 213 mothers in the intervention group, 852 mobile phone text messages were dispatched successfully from the automated system for the subsequent 4 vaccination appointments. In addition, the actual delivery status of the text message to the participants was confirmed by Ethio telecom. Out of the automatically sent 852 mobile phone text messages, 764 (89.7%) were delivered successfully to the study participants. Among the 213 participants, 176 (82.6%), 11 (5.2%), 10 (4.7%), and 7 (3.3%) received 4, 3, 2, and 1 text message reminder, respectively (Figure 2). At the end of the study, we conducted a telephone survey of the participants in the intervention group to assess the reading status of the text message reminders. We attempted to contact the 204 participants in the intervention group for whom at least one text message was delivered. Among the 204 participants, 192 participants responded to our phone call. Of the total 728 text messages sent to 192 participants, 635 (87.3%) mobile phone text message reminders were seen by the study participants.

### Study Power Analysis

For this particular trial, post hoc study power was calculated using STATA 14 software. Taking into account the superiority design of the trial, the power to detect the observed difference was 89.4% for full vaccination and 99.8% for on-time full vaccination.

## Discussion

### Principal Findings

This trial assessed the role of implementing a locally developed mobile phone text message reminder system to improve the immunization program in the Ethiopian context. The findings of our study indicated that text message reminders have a positive significant effect on the timely completion of routine vaccinations. This study also showed that text message reminders significantly improved timely vaccination uptake in the sixth week, tenth week, fourteenth week, and ninth month after childbirth for specific vaccines. In the intervention, of the automatically sent 852 text messages, 764 (89.7%) were delivered successfully to the study participants. This trial showed that a significantly higher proportion of infants in the intervention group were fully vaccinated as compared to infants in the usual care group (one-tailed *P*=.002). Based on our previous baseline assessments in the study area, where 34.5% of the caregivers reported nonattendance at child vaccination appointments owing to forgetfulness [40], text message reminders might help to track the schedules and improve attendance to child vaccination services. The full vaccination coverage at 12 months in both the intervention and usual care groups was lower than the national targets, indicating the importance of additional interventions to strengthen the immunization program. Our findings were consistent with those reported in studies from Bangladesh [66], Nigeria [68], and Côte d'Ivoire [71], which reported that SMS text reminders improved childhood vaccination coverage. Our findings were also in line with those of other studies that observed that mobile phone–based reminders significantly improved attendance to different health service appointments [61,75,99-101]. On the contrary, community-based studies from Ethiopia [75], Kenya [67], and South Africa [76] reported that the effect of SMS-based mobile phone interventions on full vaccination coverage was not statistically significant at 5% significance level. This difference can be explained by the differences in the study settings and differences in the study populations. It is also worth highlighting that the nature of the mHealth intervention packages might explain the observed differences. A systematic review conducted in an African setting also reported that the effect of one-way SMS reminders is uncertain and only some studies have shown the impact of SMS reminders on child vaccination attendance [57].

In this trial, infants from the intervention group had higher vaccination coverage for specific vaccines as compared to the infants from the usual care group. This finding is in line with that of other studies conducted in Zimbabwe [44], Kenya [43], and the United States of America [102,103]. However, randomized clinical trials conducted in the United States of America [73], Guatemala [74], Pakistan [70], and Nigeria [68] observed a higher vaccination coverage for specific vaccines attributable to text message reminders, which are not statistically significant at 5% significance level. The differences in the effect of the text message reminders could be related to the way text messages had been developed and delivered to the study participants.

In this study, we also observed that mobile phone text message reminders have a statistically significant effect on the timely completion of childhood vaccinations (one-tailed *P*<.001). This implies that text message reminders could serve as a valuable tool to track scheduled vaccination appointments in which the text reminders sent to clients one day before the due date of the scheduled vaccinations promptly notifies them to plan and vaccinate their child on time. Studies from Bangladesh [66], Australia [92], and Nigeria [68] corroborate this finding and reported that text reminders significantly improved the timely receipt of all scheduled vaccinations. A clustered randomized controlled trial conducted in rural Kenya also reported that SMS reminders coupled with incentives significantly improved the timeliness of vaccination [67]. However, this particular study observed that one-way mobile phone text message reminders without incentives have no statistically significant effect at 5% significance level on the timely vaccination of infants [67]. Methodological differences in measuring the timeliness of vaccination, delivery of the intervention package, and the study context may explain the effect size differences. In this study, mobile phone text message reminders increased the attendance of infants who received the recommended specific vaccines on time. Studies from the United States of America [104,105] and Nigeria [68] also reported similar findings.

In the subgroup analysis, this study showed that the intervention had positive effects on the subgroups of the sociodemographic characteristics of the study participants. This means that the intervention package appears to be beneficial for almost all mothers in the intervention group, implying that the proposed intervention has the potential to improve timely vaccination in the larger community. Besides, in the subgroup analysis, household wealth index was identified as a significant effect modifier with which mothers from middle or rich households were more benefited from the text message reminder intervention. This study also pointed out that using text message reminders is not without difficulties that need to be addressed. This was evidenced by findings of other studies that reported similar barriers [73,104]. As confirmed by phone calls at the end of the study, the majority (635/728, 87.3%) of the mobile phone text message reminders were seen by participants during the study period while the remaining text messages sent to mothers were not seen by the participants. In a real-world setting, this could be a common phenomenon, and our analysis considered the modified intention-to-treat analysis principle instead of the per-protocol analysis to declare the effectiveness of the mobile phone text message reminders in the routine EPI.

### Implications for Practice and Research

Our results add unique findings to the body of literature on the impact of mobile phone text message reminders on timely vaccination, which has not been studied so far in the Ethiopian context. Our findings indicated that mobile phone–based text message reminders can be added to the arsenal of other interventions as a supplement to existing immunization programs. Moreover, the results of this study could help in guiding the future adoption and implementation of mHealth interventions in low-income countries such as Ethiopia. Program managers could consider using this system to improve child immunization service uptake as the mobile phone–based reminder system for this trial was designed in Ethiopia with the local context. This study was limited to only those mothers who have mobile phones. In implementing a text messaging reminder system, the needs of those mothers who have no mobile phones need to be addressed. Mobile phone ownership may also increase as mobile services and mobile phones become increasingly ubiquitous in low-income countries. The digital health literacy of the end users might also influence the effective implementation of mHealth interventions in different contexts, which demand prior assessment in future trials. To scale up the mobile phone–based text message interventions in the national EPI of a resource-constrained setting, further studies that guide large-scale implementation are recommended.

### Strengths and Limitations

This study has the following strengths. In this trial, the study participants were allocated randomly, which resulted in balanced demographic characteristics between the study groups. This study also applied allocation concealment during the enrollment of the study participants. In addition, objective measures were used for ascertaining the vaccination status as an outcome and outcome assessors were also blind to the intervention.

Our study had the following limitations. We enrolled mothers who owned a mobile phone and presented for infant vaccination in health facilities of Gondar city, which may limit the generalizability of our findings to the general population. Owing to the nature of this study, blinding of study participants was not possible. Further, the possibility of information contamination among the participants across the study groups cannot be ruled out. The automated mobile phone text message reminder system was not set to provide information on whether mothers had received and read the text message reminders or not. Hence, the reading status of the text message reminders was confirmed at the end of the study via phone calls, which might have a possibility of recall bias.

### Conclusions

In conclusion, mobile phone text message reminders significantly improved complete and timely receipt of all recommended childhood vaccinations in the study setting. Moreover, text message reminders had a significant effect in improving timely receipt of specific vaccines provided in the sixth week, tenth week, fourteenth week, and ninth month after childbirth. Thus, text message reminders can be an additional tool to usual care for improving timely completion of childhood vaccinations in resource-limited settings. Locally developed mobile phone text message reminders as a new evolution may contribute to strengthening the routine immunization program and should be considered by policy makers and program managers to improve timely completion of vaccinations. Further rigorous interventional studies in different contexts with more focus on implementation challenges of mHealth interventions are recommended.

## Appendix

Multimedia Appendix 1 Client registration, client list, vaccination appointment scheduling, and vaccination reminder analysis pages of the automated reminder system.

Multimedia Appendix 2 Mobile text message reminder contents and reminder message sending process.

Multimedia Appendix 3 CONSORT-eHEALTH checklist (V 1.6.1).

## Abbreviations

EPI: expanded program on immunization
mHealth: mobile health
RR: risk ratio
VPD: vaccine-preventable disease
