# Supplementary figures and images for: Effect of Mobile Phone Text Message Reminders on the Completion and Timely Receipt of Routine Childhood Vaccinations: Superiority Randomized Controlled Trial in Northwest Ethiopia
Source: JMIR Mhealth Uhealth. 2021 Jun 15;9(6):e27603. doi: 10.2196/27603 (PMC8277338; doi:10.2196/27603)

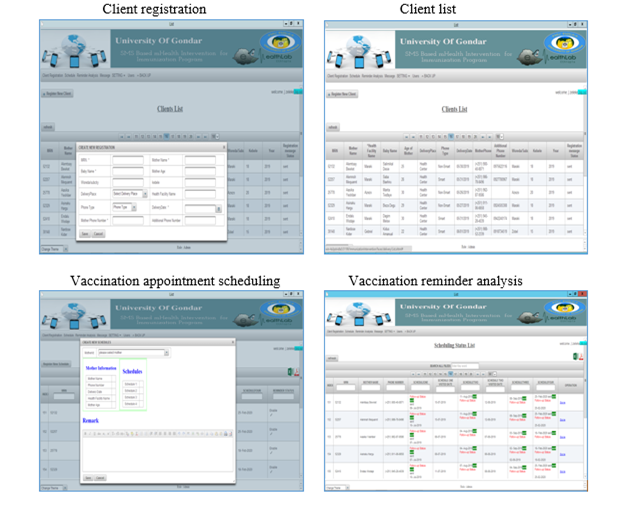

Supplement: Multimedia Appendix 1 [file mhealth_v9i6e27603_app1.png]

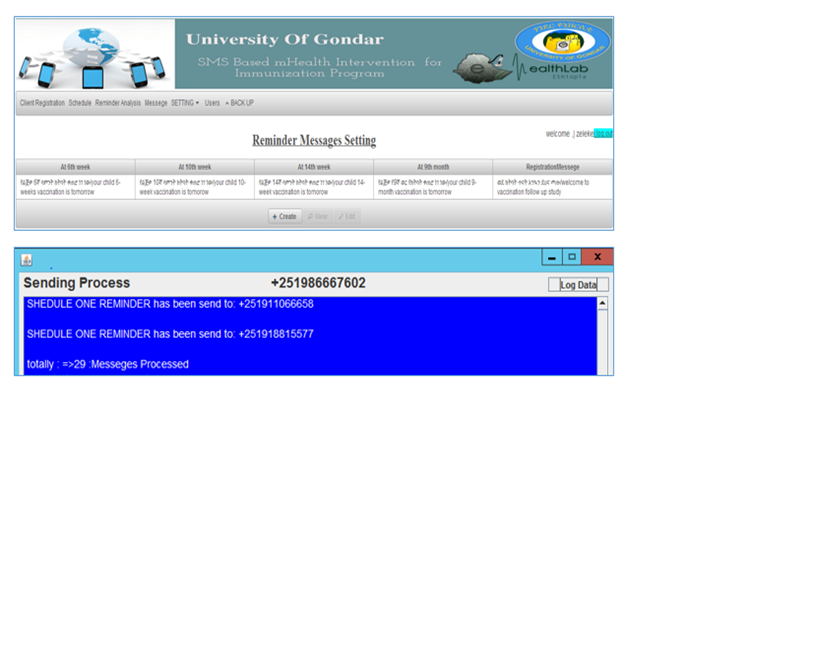

Supplement: Multimedia Appendix 2 [file mhealth_v9i6e27603_app2.png]
